# Impact of intrinsic subtype on predicting axillary lymph node metastasis in breast cancer

**DOI:** 10.3892/ol.2014.2333

**Published:** 2014-07-10

**Authors:** MASATAKA SAWAKI, AI IDOTA, MARI ICHIKAWA, NAOMI GONDO, AKIYO HORIO, NAOTO KONDO, MASAYA HATTORI, TAKASHI FUJITA, YASUSHI YATABE, HIROJI IWATA

**Affiliations:** 1 Department of Breast Oncology, Aichi Cancer Center Hospital, Nagoya, Aichi 464-8681, Japan; 2 Department of Pathology and Molecular Diagnostics, Aichi Cancer Center Hospital, Nagoya, Aichi 464-8681, Japan

**Keywords:** breast cancer, intrinsic subtype, lymph node metastasis, tumor size

## Abstract

Axillary lymph node (LN) metastasis is one of the most important prognostic factors for the survival of breast cancer. The correlation between LN metastasis and the tumor (T) category has previously been investigated in certain case series. At present, the initial treatment approach is to define the intrinsic subtype, as it is significant in determining medical treatments, as well as being a prognostic factor. However, the intrinsic subtype is not known to predict the frequency of LN metastasis. The aim of the present study was to evaluate the frequency of LN metastasis with regard to tumor size according to the intrinsic subtype. In total, 654 patients with primary breast cancer were evaluated who underwent surgical resection between 2010 and 2011 at the Aichi Cancer Center Hospital (Nagoya, Aichi). The clinical and pathological data were analyzed for patients who underwent an axillary LN dissection or a sentinel LN biopsy for primary breast cancer. The intrinsic subtype of the primary tumors was classified using immunohistochemical staining of thin, paraffin-embedded sections. In total, 157 (24.0%) of the 654 patients exhibited LN metastasis, and according to the primary tumor category, a larger tumor size was found to correlate with a higher proportion of LN positivity, as well as with the luminal A subtypes (n=364). In luminal B subtypes (n=110), T1a (n=2), T1b (n=12), T1c (n=55), T2 (n=34), and T3 (n=2) exhibited 50, 8.3, 38.2, 55.9 and 50% LN positivity, respectively. In luminal-human epidermal growth factor receptor 2 (HER2) subtypes (n=46), T1c (n=17), T2 (n=10), and T3 (n=1) exhibited 40.1, 60 and 100% LN positivity, respectively. In HER2 subtypes (n=53), T1a (n=6), T1b (n=4), T1c (n=15), and T2 (n=10) exhibited 16.7, 25, 46.7 and 60% LN positivity, respectively. In triple-negative subtypes (n=81), T1b (n=15), T1c (n=29), T2 (n=20), and T3 (n=2) exhibited 26.7, 24.1, 50 and 50% LN positivity, respectively. In conclusion, the intrinsic subtype is significant in predicting the frequency of LN metastasis with regard to tumor size.

## Introduction

Breast cancer is the most common type of malignancy in females, accounting for 25% of all female cases and resulting in >1.67 million cases worldwide in 2012. It is also the most common cause of cancer-related mortality in females with ~522,000 mortalities in 2012 (1). For the treatment of breast cancer, identification of the intrinsic subtype is important, as well as the anatomical staging (2). Axillary lymph node (LN) metastasis, which indicates established cancerous dissemination, is one of the most important prognostic factors for the survival of breast cancer (3). It is a multifactorial event determined by the patient and tumor characteristics. The correlation between LN metastasis and T category has previously been investigated in a certain case series (4). At present, the initial treatment approach is to define the intrinsic subtype, as it is significant in determining medical treatments, as well as being a prognostic factor (2). However, the intrinsic subtype is not known to predict the frequency of LN metastasis. The aim of the present study was to evaluate the frequency of LN metastasis with regard to tumor size according to intrinsic subtype at the Aichi Cancer Center Hospital (Nagoya, Aichi).

## Patients and methods

### Patient characteristics and procedures

**Figure 1:**
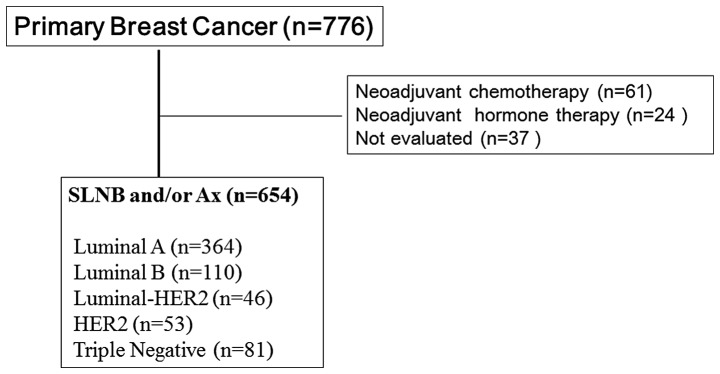
Consolidated Standards of Reporting Trials diagram. Patients with primary breast cancer, who underwent surgical resection between 2011 and 2012 at the Aichi Cancer Center (Nagoya, Japan) were included in the present study. Patients who had received neoadjuvant medical treatment and those who had not undergone a lymph node biopsy were excluded. The subtypes were classified as follows: Luminal A, ER- and/or PgR-positive, HER2-negative and Ki-67 of ≤20%; luminal B, ER- and/or PgR-positive, HER2-negative and Ki-67 of >20%; luminal-HER2, ER- and/or PgR-positive and HER2-positive; HER2, ER- and PgR-negative, and HER2-positive; and triple-negative, ER-, PgR- and HER2-negative. SLNB, sentinel lymph node biopsy; Ax, axillary dissection; ER, estrogen receptor; PgR, progesterone receptor; HER2, human epidermal growth factor receptor 2.

A total of 776 patients with primary breast cancer who underwent surgical resection between 2010 and 2011 at the Aichi Cancer Center were included in the present study. A Consolidated Standards of Reporting Trials diagram is shown in Fig. 1 to avoid patient bias in this retrospective study. Patients who received neoadjuvant medical treatment and patients who had not undergone an LN biopsy were excluded. Therefore, the clinical and pathological data were analyzed for 654 patients who underwent an axillary LN dissection or sentinel LN (SLN) biopsy for primary breast cancer. In clinically LN-negative cases, an SLN biopsy was performed. The SLN was bisected along its major axis and a diagnosis was determined from frozen sections obtained during surgery. If the SLN indicated LN positivity, patients were treated with a standard level one and two axillary dissection. Clinically LN-positive (i.e. cytologically positive) patients underwent an axillary dissection. The SLN section was routinely stained with hematoxylin and eosin for examination. Patients provided written informed consentand the study was approved by the ethics committee of Aichi Cancer Center.

### Extent of the primary tumor

Lesions were identified preoperatively by palpation, measured using ultrasound and the largest diameter was recorded as the T category according to the tumor, necrosis, metastasis classification (UICC, Sixth Edition, 2002) (5). The tumors were categorized as follows: Tis, carcinoma *in situ*; T1mic, microinvasion of ≤0.1 cm; T1a, tumor of >0.1 to ≤0.5 cm; T1b, tumor of >0.5 to ≤1 cm; T1c, tumor of >1 to ≤2 cm; T2, tumor of >2 to ≤5 cm; T3, tumor of >5 cm; and T4, any size with direct extension to the chest wall or skin. When the primary tumor could not be assessed or when there was no evidence of a primary tumor, the clinical T size was determined as Tis.

### Clinicopathological definition of intrinsic subtype

The intrinsic subtypes of primary tumors were classified by immunohistochemical (IHC) examination of paraffin-embedded thin sections as follows: Luminal A subtype, estrogen receptor (ER)- and/or progesterone receptor (PgR)-positive, human epidermal growth factor receptor 2 (HER2)-negative and Ki-67 of ≤20%; luminal B subtype, ER- and/or PgR-positive, HER2-negative and Ki-67 of >20%; luminal-HER2 subtype, ER- and/or PgR-positive and HER2-positive; HER2 subtype, ER-negative, PgR-negative and HER2-positive; and triple-negative subtype, ER-, PgR- and HER2-negative. In the proposed classification, the Ki-67 labeling index was particularly significant for distinguishing between the luminal A and B (HER2-negative) subtypes (2).

### IHC assessment

The expression of ER and PgR was scored using the Allred score (6–8). In brief, the following proportion score was assigned, which represented the estimated proportion of positively stained tumor cells: 0, None; 1, <1/100; 2, 1/100 to 1/10; 3, 1/10 to 1/3; 4, 1/3 to 2/3; and 5, >2/3. Next, the following intensity score was assigned, which represented the average intensity of positive tumor cells as follows: 0, None; 1, weak; 2, intermediate; and 3, strong. The sum of the proportion and intensity scores provided the total score, which ranged between 0 and 8 (7). The scores were regarded as positive if the total score was >3. HER2 was considered to be positive if a score of 3+ (uniform, intense membrane staining of >30% of invasive tumor cells) was obtained according to the manufacturer’s instructions for the HercepTest^®^ (Dako, Carpinteria, CA, USA). Fluorescence *in situ* hybridization was performed when a score of 2+ was obtained and gene amplification was assessed. The ratio of HER2 to chromosome 17 centromere was ≥2.2, which was considered to be positive (9). The Ki-67 expression was quantified using a visual grading system. The percentage of Ki-67-positive cells among the total number of counted neoplastic cells was determined at a magnification of ×400 using an eye-piece graticule (BX51^®^; Olympus Corporation, Tokyo, Japan) at areas in which Ki67 staining was particularly prevalent (10,11) An estimated percentage of Ki-67-positive cells was determined, and scored according to two categories that were organized by increasing percentage intervals of 0–20% and >20%. For the present study, >20% of Ki-67-positive cells was regarded as positive (10). The slides were scored independently by two pathologists.

## Results

### Patients and intrinsic subtype

A total of 654 patients were included in the present study and the patient characteristics are demonstrated in Table I. Of the 654 patients, 157 patients (24.0%) exhibited LN metastasis. The proportion of intrinsic subtypes in the present study was as follows: Luminal A, n=364 (55.7%); luminal B, n=110 (16.8%); luminal-HER2, n=46 (7.0%); HER2, n=53 (8.1%); and triple-negative, n=81 (12.4%).

### Lymph node positivity by T category in all patients

**Figure 2:**
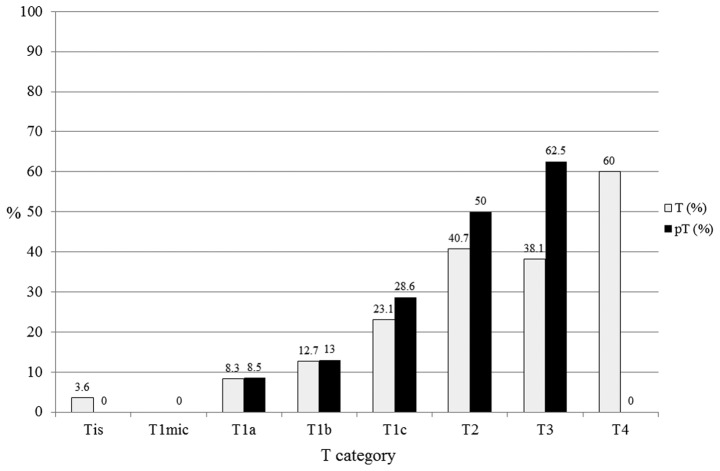
Lymph node (LN) positivity according to the tumor size category of all patients (n=654). In all patients, pT1a (n=47), pT1b (n=108), pT1c (n=241), pT2 (n=130) and pT3 (n=8) exhibited 8.5, 13.0, 28.6, 50.0 and 62.5% LN positivity, respectively. Larger tumor size correlated with a higher proportion of LN positivity. T, tumor; pT, primary tumor; Tis, carcinoma *in situ*; T1mic, microinvasion of ≤0.1 cm; T1a, tumor of >0.1 to ≤0.5 cm; T1b, tumor of >0.5 to ≤1 cm; T1c, tumor of >1 to ≤2 cm; T2, tumor of >2 to ≤5 cm; T3, tumor of >5 cm; T4, any size with direct extension to the chest wall or skin.

LN positivity was evaluated according to T category in all patients (n=654; Fig. 2). In all patients, pT1a (n=47), pT1b (n=108), pT1c (n=241), pT2 (n=130) and pT3 (n=8) exhibited 8.5, 13, 28.6, 50.0 and 62.5% LN positivity, respectively. A larger tumor size was found to correlate with a higher proportion of LN positivity with regard to the pT category.

### Lymph node positivity by T category in each subtype

**Figure 3:**
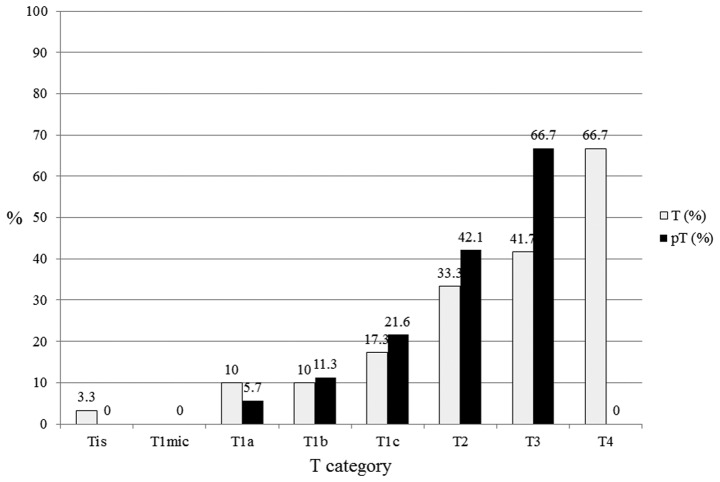
In luminal A subtypes (n=364), pT1a (n=35), pT1b (n=71), pT1c (n=125), pT2 (n=56) and pT3 (n=3) exhibited 5.7, 11.3, 21.6, 42.1 and 66.7% LN positivity, respectively. Larger tumor size correlated with a higher proportion of lymph node positivity. T, tumor; pT, primary tumor; Tis, carcinoma *in situ*; T1mic, microinvasion of ≤0.1 cm; T1a, tumor of >0.1 to ≤0.5 cm; T1b, tumor of >0.5 to ≤1 cm; T1c, tumor of >1 to ≤2 cm; T2, tumor of >2 to ≤5 cm; T3, tumor of >5 cm; T4, any size with direct extension to the chest wall or skin.

**Figure 4:**
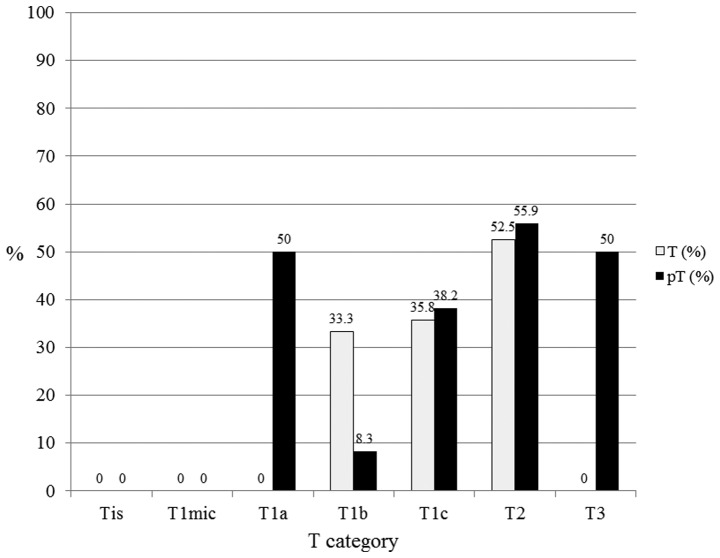
In luminal B subtypes (n=110), pT1a (n=2), pT1b (n=12), pT1c (n=55), pT2 (n=34) and pT3 (n=2) exhibited 50, 8.3, 38.2, 55.9 and 50% lymph node positivity, respectively. T, tumor; pT, primary tumor; Tis, carcinoma *in situ*; T1mic, microinvasion of ≤0.1 cm; T1a, tumor of >0.1 to ≤0.5 cm; T1b, tumor of >0.5 to ≤1 cm; T1c, tumor of >1 to ≤2 cm; T2, tumor of >2 to ≤5 cm; T3, tumor of >5 cm.

**Figure 5:**
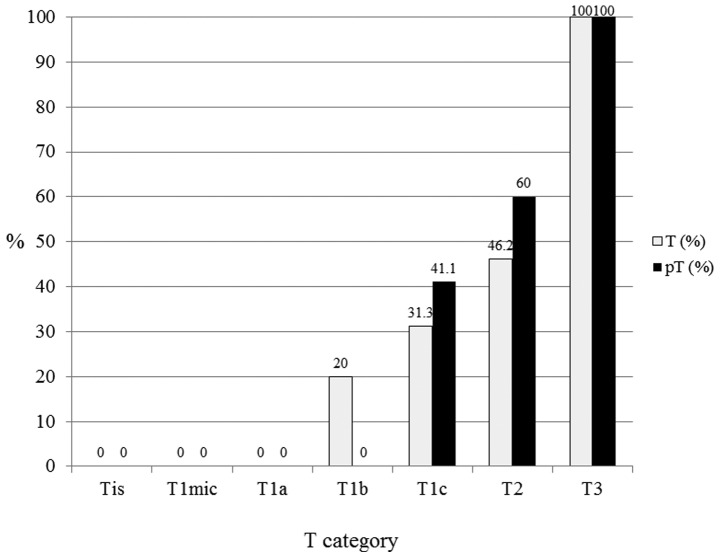
In human epidermal growth factor receptor 2-luminal subtypes (n=46), pT1c (n=17), pT2 (n=10) and pT3 (n=1) exhibited 41.1, 60 and 100% lymph node positivity, respectively. T, tumor; pT, primary tumor; Tis, carcinoma *in situ*; T1mic, microinvasion of ≤0.1 cm; T1a, tumor of >0.1 to ≤0.5 cm; T1b, tumor of >0.5 to ≤1 cm; T1c, tumor of >1 to ≤2 cm; T2, tumor of >2 to ≤5 cm; T3, tumor of >5 cm.

**Figure 6:**
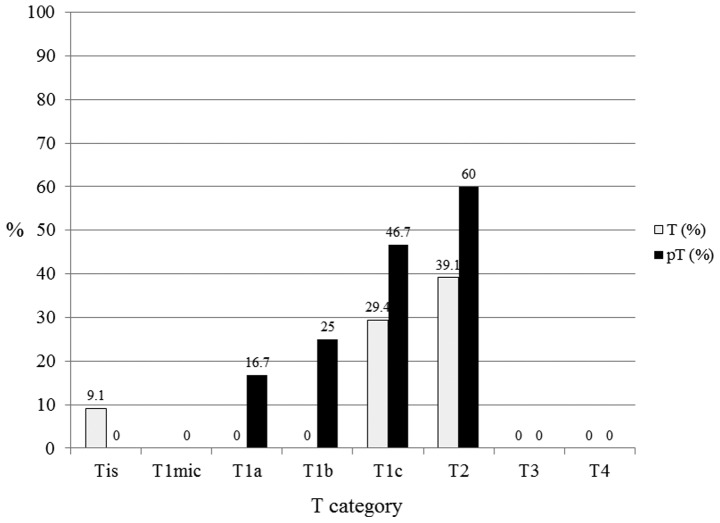
In human epidermal growth factor receptor 2 subtypes (n=53), pT1a (n=6), pT1b (n=4), pT1c (n=15) and pT2 (n=10) exhibited 16.7, 25, 46.7 and 60% lymph node positivity, respectively. T, tumor; pT, primary tumor; Tis, carcinoma *in situ*; T1mic, microinvasion of ≤0.1 cm; T1a, tumor of >0.1 to ≤0.5 cm; T1b, tumor of >0.5 to ≤1 cm; T1c, tumor of >1 to ≤2 cm; T2, tumor of >2 to ≤5 cm; T3, tumor of >5 cm; T4, any size with direct extension to the chest wall or skin.

**Figure 7:**
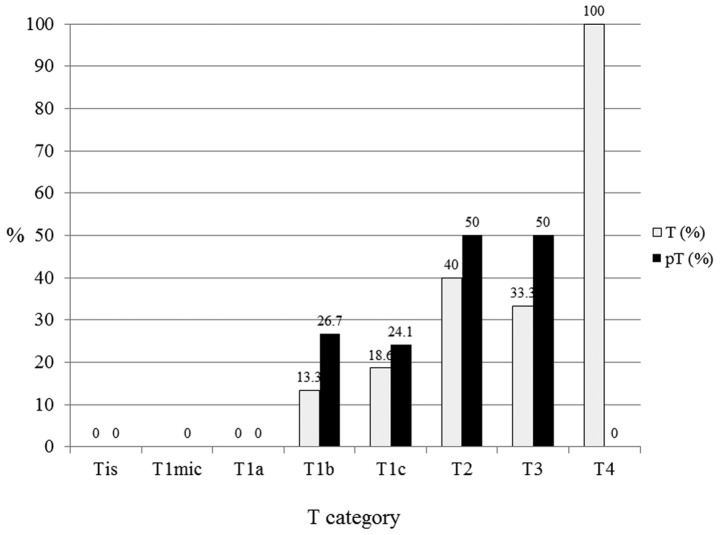
In triple-negative subtypes (n=81), pT1b (n=15), pT1c (n=29), pT2 (n=20) and pT3 (n=2) exhibited 26.7, 24.1, 50 and 50% lymph node positivity, respectively. T, tumor; pT, primary tumor; Tis, carcinoma *in situ*; T1mic, microinvasion of ≤0.1 cm; T1a, tumor of >0.1 to ≤0.5 cm; T1b, tumor of >0.5 to ≤1 cm; T1c, tumor of >1 to ≤2 cm; T2, tumor of >2 to ≤5 cm; T3, tumor of >5 cm; T4, any size with direct extension to the chest wall or skin.

In the luminal A subtype (n=364; Fig. 3), pT1a (n=35), pT1b (n=71), pT1c (n=125), pT2 (n=56) and pT3 (n=3) exhibited 5.7, 11.3, 21.6, 42.1 and 66.7% LN positivity, respectively. Larger tumor size was found to correlate with a higher proportion of LN positivity. In the luminal B subtypes (n=110), pT1a (n=2), pT1b (n=12), pT1c (n=55), pT2 (n=34), and pT3 (n=2) exhibited 50.0, 8.3, 38.2, 55.9 and 50.0% LN positivity, respectively (Fig. 4). In the luminal-HER2 subtypes (n=46), pT1c (n=17), pT2 (n=10) and pT3 (n=1) exhibited 41.1, 60.0 and 100.0% LN positivity, respectively (Fig. 5). In the HER2 subtypes (n=53), pT1a (n=6), pT1b (n=4), pT1c (n=15), and pT2 (n=10) exhibited 16.7, 25, 46.7 and 60.0% LN positivity, respectively (Fig. 6). In the triple-negative subtypes (n=81), pT1b (n=15), pT1c (n=29), pT2 (n=20) and pT3 (n=2) exhibited 26.7, 24.1, 50.0 and 50.0% LN positivity, respectively (Fig. 7).

## Discussion

To the best of our knowledge, this is the first report to investigate the likelihood of LN metastasis according to intrinsic subtype and tumor size. The intrinsic subtypes of breast cancer have, however, previously been analyzed by gene expression arrays (12–14). These subtypes exhibit different epidemiological risk factors (15), natural histories (16), and responses to systemic and local therapies (17). These differences imply that clinicians managing breast cancer are required to consider cases according to the various, distinct subtypes in order to properly assess the relevant evidence and determine an appropriate therapeutic strategy (2). As it is not always feasible to obtain gene expression array data, a simplified clinical classification has been adopted for performance in the clinical setting (2,18). The subtypes defined by clinicopathological criteria are similar, although not identical, to the intrinsic subtypes and represent a convenient approximation (2).

Axillary LN metastasis is one of the most important prognostic factors. However, prior to performing an SLN biopsy or axillary dissection, it is not possible to determine whether the patient is LN-negative. There are few studies that have been reported to predict the frequency of LN metastasis according to intrinsic subtype (19,20), however, the correlation between LN metastasis and T category has been investigated in certain case series (4). Multivariate predictive models have been produced that incorporate tumor size, patient age, S phase and PgR as independent predictors (21), however, these were not classified by intrinsic subtype. The risk of LN metastasis is considered to be significantly higher for palpable breast tumors (4), thus, clinical tumor size is important for predicting LN metastasis. The likelihood of LN metastasis may be predicted following a preoperative core needle biopsy using IHC examination.

A limitation of the current study was that the intrinsic subtype should have originally been identified by gene expression array; however, a simplified clinicopathological classification system was used in the clinical setting (2). Local quality control of Ki-67 staining was considered to be a significant factor in the present study. However, during IHC examination, the cut-off for the percentage of Ki-67-positivity, for distinguishing between luminal A and B, was controversial as it has been identified to be between 14 (18) and 20% (10). In the current study, few patients with specific intrinsic subtypes were available for analysis. Few patients exhibited stage T4 tumors, as almost all patients who had undergone neoadjuvant medical treatment were excluded. Neoadjuvant medical treatment is recommended to patients prior with stage T4 tumors (22).

In conclusion, the intrinsic subtype and tumor size are important for predicting the frequency of LN metastasis in breast cancer patients. By knowing the intrinsic subtype and tumor size LN metastasis may be predicted prior to surgery.

## Figures and Tables

**Table I:** Patient characteristics.

Variable	Subjects	Lymph node positivity
Total, n (%)	654 (100)	157 (24.0)
Age, years		
Median	54.5	-
Range	20–85	-
Clinical T stage, n (%)		
Tis	83 (12.7)	3 (3.6)
T1a	12 (1.8)	1 (8.3)
T1b	110 (16.8)	14 (12.7)
T1c	251 (38.4)	58 (23.1)
T2	172 (26.3)	70 (40.7)
T3	21 (3.2)	8 (38.1)
T4	5 (0.8)	3 (60.0)
Pathological T stage, n (%)		
Tis	107 (16.4)	0 (0.0)
T1mic	13 (2.0)	0 (0.0)
T1a	47 (7.2)	4 (8.5)
T1b	108 (16.5)	14 (13)
T1c	241 (36.9)	69 (28.6)
T2	130 (19.9)	65 (49.6)
T3	8 (1.2)	5 (62.5)
T4	0 (0.0)	0 (0.0)
Intrinsic subtype, n (%)		
Luminal A	364 (55.7)	63 (17.3)
Luminal B	110 (16.8)	43 (39.1)
HER2-luminal	46 (7.0)	14 (30.4)
HER2	53 (8.1)	15 (28.3)
Triple-negative	81 (12.4)	22 (27.2)
Histological classification, n (%)		
Non-invasive carcinoma	107 (16.4)	0 (0.0)
Papillotubular carcinoma	169 (25.8)	44 (26.0)
Solid-tubular carcinoma	57 (8.7)	22 (38.6)
Scirrhous carcinoma	260 (39.8)	81 (31.2)
Special types	61 (9.3)	10 (16.4)

Tis, carcinoma *in situ*; T1mic, microinvasion of ≤0.1 cm; T1a, tumor of >0.1 to ≤0.5 cm; T1b, tumor of >0.5 to ≤1 cm; T1c, tumor of >1 to ≤2 cm; T2, tumor of >2 to ≤5 cm; T3, tumor of >5 cm; T4, any size with direct extension to the chest wall or skin; HER2, human epidermal growth factor receptor 2.
